# Long-chain bases of sphingolipids are transported into cells via the acyl-CoA synthetases

**DOI:** 10.1038/srep25469

**Published:** 2016-05-03

**Authors:** Tomomi Narita, Tatsuro Naganuma, Yurie Sase, Akio Kihara

**Affiliations:** 1 Laboratory of Biochemistry, Faculty of Pharmaceutical Sciences, Hokkaido University, Kita 12-jo, Nishi 6-chome, Kita-ku, Sapporo 060-0812, Japan

## Abstract

Transport of dietary lipids into small-intestinal epithelial cells is pathologically and nutritionally important. However, lipid uptake remains an almost unexplored research area. Although we know that long-chain bases (LCBs), constituents of sphingolipids, can enter into cells efficiently, the molecular mechanism of LCB uptake is completely unclear. Here, we found that the yeast acyl-CoA synthetases (ACSs) Faa1 and Faa4 are redundantly involved in LCB uptake. In addition to fatty acid-activating activity, transporter activity toward long-chain fatty acids (LCFAs) has been suggested for ACSs. Both LCB and LCFA transports were largely impaired in *faa1*Δ *faa4*Δ cells. Furthermore, LCB and LCFA uptakes were mutually competitive. However, the energy dependency was different for their transports. Sodium azide/2-deoxy-D-glucose treatment inhibited import of LCFA but not that of LCB. Furthermore, the ATP-AMP motif mutation *FAA1 S271A* largely impaired the metabolic activity and LCFA uptake, while leaving LCB import unaffected. These results indicate that only LCFA transport requires ATP. Since ACSs do not metabolize LCBs as substrates, Faa1 and Faa4 are likely directly involved in LCB transport. Furthermore, we revealed that ACSs are also involved in LCB transport in mammalian cells. Thus, our findings provide strong support for the hypothesis that ACSs directly transport LCFAs.

Cells absorb a variety of extracellular substances through the plasma membrane and utilize them as energy sources or precursors for biomolecules. Since the hydrophobic barrier formed by the lipid bilayers does not allow transport of hydrophilic materials, cells must import them via specific transporters. However, there is still no conclusion whether hydrophobic materials such as fatty acids (FAs) enter into cells under physiological conditions via passive diffusion or via transporters.

Expression cloning experiments have identified *FATP1* (FA transporter protein 1) and *ACSL1* [acyl-CoA synthetase long-chain (ACSL) family member 1] to enhance uptake of long-chain FAs (LCFAs; C11–C20) when cDNA of each was introduced into cultured cells^1^. ACSL1 belongs to the acyl-CoA synthetase (ACS) family, whose members catalyze activation of FAs, *i.e.* conversion of FAs to acyl-CoAs. Later, it turned out that FATP1 is another member of the ACS family [alias name, ACSVL5; ACS very-long-chain (ACSVL) family member 5]^2^,^3^. *In vitro* analysis has demonstrated that FATP1 indeed has ACS activity toward very-long-chain FAs (VLCFAs; ≥C21)^4^. Mammals have 26 ACS family members, which are divided into 6 subfamilies according to substrate specificity and sequence similarity: ACS short-chain (ACSS), ACS medium-chain (ACSM), ACSL, ACSVL, ACS bubblegum (ACSBG), and ACS family (ACSF)^2^. Yeast has 7 ACS family members: Faa1–4, Fat1, 2, and Acs1^5^. Among them, Faa1 exhibits the highest ACS activity toward LCFAs^6^, while Faa4 has redundant ACS activity with Faa1. Double deletion of *FAA1* and *FAA4* genes (*faa1*Δ *faa4*Δ) causes ≥95% reduction in ACS activity toward oleic acid and greatly reduces transport of LCFAs into cells^6^. Fat1, which shares the highest sequence similarity with mammalian FATP1 among yeast ACSs, exhibits ACS activity toward VLCFAs^7^.

So far, FA transport activities of ACS family members have not been verified experimentally using purified proteins due to technical difficulties inherent in such *in vitro* assays, leading to debates whether ACSs function only in FA activation or both in FA activation and FA transport^5^,^8^. In the former case, ACSs would cause an apparent increase in LCFA uptake by trapping LCFAs as acyl-CoAs so they do not diffuse back to the extracellular spaces or by decreasing the intracellular LCFA concentration. In the latter case, the transport and activation of LCFAs by ACSs would be coupled, a process called vectorial acylation.

Sphingolipids are one of the major lipid components of eukaryotic membranes and have a wide range of physiological functions, including cell adhesion, skin permeability barrier formation, myelin maintenance, immunity, spermatogenesis, and glucose metabolism^9^,^10^,^11^,^12^,^13^,^14^,^15^. Ceramide, the hydrophobic backbone of sphingolipids, is composed of a FA and a long-chain base (LCB). The most abundant LCB in mammals is sphingosine (SPH), which contains a *trans* double bond between the C4 and C5 positions (Fig. 1a). SPH and the saturated LCB dihydrosphingosine (DHS) exist ubiquitously among mammalian tissues. Yeast does not have SPH but instead contains DHS and phytosphingosine (PHS), which possesses a hydroxyl group at the C4 position^16^ (Fig. 1a). In mammals, PHS exists in specific tissues, such as the skin, small intestine, and kidney^9^.

Eukaryotes can utilize not only endogenous LCBs produced via *de novo* biosynthetic pathways but also exogenous LCBs for sphingolipid synthesis. For example, food contains abundant sphingolipids: per capita sphingolipid consumption is estimated to be 300–400 mg per day^17^. Dietary sphingolipids are degraded to FAs and LCBs before absorption into small-intestinal epithelial cells, since complex sphingolipids and ceramides scarcely enter into cells^18^. Beneficial effects of dietary sphingolipids have been reported, such as reduction in serum low-density lipoprotein (LDL) cholesterol levels, prevention of colon carcinogenesis and inflammation, and improved skin barrier function^19^,^20^,^21^,^22^,^23^. Plasma sphingolipids, especially SPH 1-phosphate (S1P), are other exogenous sources of LCBs. S1P is a lipid mediator and causes several cellular responses such as proliferation, activation or inhibition of cell migration, and adherens junction assembly^9^. S1P exists in plasma at levels of several hundred nanomolar^9^. However, S1P itself hardly enters into cells: it is dephosphorylated by cell surface lipid phosphate phosphatase to SPH, which then enters into endothelial cells^24^. At present, however, the molecular mechanism of the LCB transport is completely unclear.

Not all lipids can be transported into cells: rather, only a few classes of lipids—including LCFAs, LCBs, and monoacylglycerols—can do so efficiently. Uptake efficiencies of other classes of lipids—such as complex sphingolipids, ceramides, S1P, triacylglycerol, and glycerophospholipids—are low. Although natural ceramides and glycerophospholipids with LCFAs are hardly imported into cells as is, shortening the FA moiety allows them (*i.e.*, artificial short-chain ceramides and glycerophospholipids) to enter. Furthermore, the transport efficiencies of saturated LCFAs such as palmitic acid and stearic acid are high, while those of saturated VLCFAs such as arachidic acid and behenic acid are low^25^. Thus, we can conclude that lipids that can efficiently enter into cells share a common structure: one long-chain hydrophobic backbone and a small polar head group.

We hypothesized that a structural factor limiting certain lipid transport into cells is the substrate specificity of a common transporter(s). Therefore, we examined involvement of ACSs in LCB uptake using genetically tractable yeast. We found that LCB uptake was greatly reduced in an ACS double deletion mutant (*faa1*Δ *faa4*Δ). In addition, transports of LCFA and LCB were mutually competitive. These results suggest that Faa1 and Faa4 act as transporters both for LCFAs and LCBs. The difficulty in dissociating LCFA transport from metabolic activity (CoA addition) has created the prolonged debate for the role of ACSs in LCFA uptake, as described above. However, this debate does not apply to LCBs: they do not possess a carboxyl group, the acceptor for CoA, and so ACS-catalyzed activation step cannot take place on LCBs. Our results clearly dissociate the transport and ACS activities for LCBs and indicate that ACSs do have transport function. Furthermore, we found that members of the ACSL family—the mammalian homologs of the ACSs Faa1 and Faa4—are involved in LCB transport in mammals.

## Results

### The ACSs Faa1 and Faa4 are involved in LCB uptake

**Figure 1:**
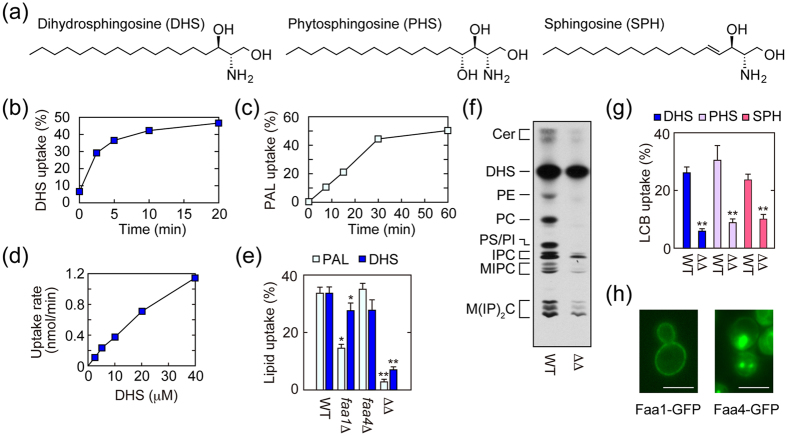
Faa1 and Faa4 are involved in the import of LCBs. (**a**) Structures of DHS, SPH, and PHS. (**b**–**e**,**g**) BY4741 (wild-type; WT) cells were incubated with [^3^H]DHS [(**b**), 20 μM; (**d**), indicated concentration] or [^3^H]palmitic acid (PAL) [(**c**), 20 μM] for the indicated time period (**b**,**c**) or for 5 min (**d**). BY4741, 6477 (*faa1*Δ), 833 (*faa4*Δ), and AOY13 (*faa1*Δ *faa4*Δ; ΔΔ) cells were labeled with 20 μM [^3^H]palmitic acid for 30 min or 20 μM [^3^H]DHS for 5 min (**e**). BY4741 and AOY13 cells were labeled with 20 μM [^3^H]DHS, [^3^H]SPH, or [^3^H]PHS for 5 min (**g**). Radioactivities associated with cells, medium, and glass test tubes were counted by a liquid scintillation counter, and those associated with cells are expressed as a percentage of the total radioactivity. Values represent the means ± SDs of three independent experiments, and statistically significant differences are indicated (*t*-test; *p < 0.05; **p < 0.01) (**e**,**g**). (**f**) BY4741 and AOY13 cells were labeled with 0.2 μCi [^3^H]DHS for 60 min. Lipids were extracted, separated by TLC, and detected by autoradiography. Cer, ceramide; PE, phosphatidylethanolamine; PC, phosphatidylcholine; PS, phosphatidylserine; PI, phosphatidylinositol; IPC, inositol phosphorylceramide; MIPC, mannosylinositol phosphorylceramide; M(IP)_2_C, mannosyldiinositol phosphorylceramide. (**h**) TNY42 (*FAA1-GFP*) and TNY51 (*FAA4-GFP*) cells were subjected to fluorescence microscopic observation using a DM5000B microscope (Leica Microsystems, Wetzlar, Germany). Bar, 4 μm.

We first examined the time course of [^3^H]DHS uptake and compared it with that of [^3^H]palmitic acid. Import of DHS was rapid and started to plateau around 5 min (Fig. 1b). The transport of palmitic acid was slower than that of DHS, and increased linearly up to 30 min (Fig. 1c). With these results in mind, we set 5 min and 30 min as the respective incubation periods for the [^3^H]DHS and [^3^H]palmitic acid uptake assays in further analyses. We next examined the uptake rate for [^3^H]DHS at various concentrations. Its uptake rate increased almost linearly up to 40 μM (Fig. 1d).

Based on the structural similarity between LCB and LCFA, *i.e.* one long-chain backbone with a small head group, we examined the possibility that ACSs, well known to facilitate LCFA uptake, are involved in LCB uptake as well. Yeast Faa1 and Faa4 have redundant function in LCFA import^6^. Therefore, we subjected single (*faa1*Δ or *faa4*Δ) or double deletion (*faa1*Δ *faa4*Δ) mutants to lipid uptake assays using [^3^H]palmitic acid (control) and [^3^H]DHS. Consistent with a previous report^6^, deletion of the *FAA1* gene reduced palmitic acid import (Fig. 1e). Double deletion of *FAA1* and *FAA4* genes further reduced the uptake (8.5% of wild-type cells), although single deletion of *FAA4* had no effect. Deletion of another yeast ACS gene, *FAT1*, had little effect on palmitic acid import in either wild type or *faa1*Δ *faa4*Δ cells (Supplementary Fig. 1a). We found that DHS uptake was also controlled by Faa1 and Faa4. The amount of imported DHS was slightly lower in *faa1*Δ cells compared to wild-type cells. However, *faa1*Δ *faa4*Δ cells suffered a much larger decrease, with the amount of imported DHS becoming a mere 21% of that in wild-type cells (Fig. 1e). Single deletion of *FAA4* or *FAT1* had little effect on DHS uptake (Supplementary Fig. 1b). The import activity of *faa1*Δ *faa4*Δ *fat1*Δ triple deletion mutants was indistinguishable from that of *faa1*Δ *faa4*Δ cells. The residual transport activity in *faa1*Δ *faa4*Δ *fat1*Δ cells may be due to the activity of other ACSs (Faa2, Faa3, Fat2, and Acs1) or to passive diffusion.

In the above lipid transport assay, we separated cells and medium by centrifugation and measured radioactivity in each fraction, and transport activity was calculated as radioactivity in the cell fraction per total radioactivities. However, this method did not allow us to discriminate between lipids truly imported into cells and those merely attached to cell surfaces (*i.e.*, not transported through plasma membrane) in the cell fraction. Therefore, we extracted lipids from cell fractions of [^3^H]DHS-labeled wild-type and *faa1*Δ *faa4*Δ cells and separated them by TLC. Since DHS metabolism occurs only within cells, DHS metabolite levels were considered to represent the amount of DHS that was genuinely imported. In wild-type cells, DHS was metabolized both to sphingolipids (ceramide, inositol phosphorylceramide, mannosylinositol phosphorylceramide, and mannosyldiinositol phosphorylceramide) and glycerophospholipids (phosphatidylethanolamine, phosphatidylcholine, phosphatidylserine, and phosphatidylinositol) (Fig. 1f). In the latter pathway, DHS was metabolized to FAs, converted to acyl-CoAs by the ACSs Faa1 and Faa4, and then incorporated into glycerophospholipids^26^,^27^. Therefore, DHS was metabolized only to sphingolipids in *faa1*Δ *faa4*Δ cells (Fig. 1f), corroborating a previous report^26^. The labeled sphingolipid levels in *faa1*Δ *faa4*Δ cells were ~25% of those in wild-type cells, which amount well correlated with that obtained from the lipid uptake assay (Fig. 1e). Thus, the DHS transport was indeed reduced in *faa1*Δ *faa4*Δ cells, and the usefulness of the lipid uptake assay was verified.

We next examined the involvement of Faa1 and Faa4 in the transports of two other LCBs, PHS and SPH, into cells. DHS and PHS are naturally occurring LCBs in yeast. On the other hand, SPH, which is the major LCB in mammals, does not exist in yeast, but they can import and metabolize it if exogenously added^26^,^28^,^29^. We found that not only the uptake of DHS but also those of SPH and PHS were reduced in *faa1*Δ *faa4*Δ cells (Fig. 1g). These results indicate that Faa1 and Faa4 are involved in the uptakes of all types of LCBs examined.

If Faa1 and Faa4 function in LCB uptake as lipid transporters, they should be localized in the plasma membrane. To test this hypothesis, we examined the localization of Faa1 and Faa4 chromosomally fused with GFP by microscopic observation. We found that Faa1-GFP was mainly localized in the plasma membrane (Fig. 1h). Faa4-GFP was also localized in the plasma membrane but only partly; most of it was localized in internal organelles. The partial localization of Faa4 in the plasma membrane is consistent with the weak transport activity of Faa4 (Fig. 1e).

### Decreased LCB uptake in *faa1*Δ *faa4*Δ cells is not caused by increased LCB efflux or changed lipid composition

**Figure 2:**
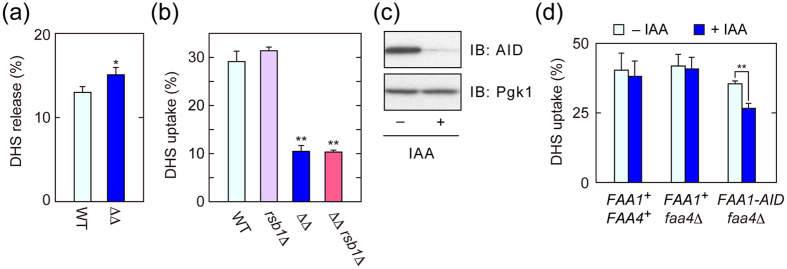
Decreased LCB uptake in *faa1*Δ *faa4*Δ cells is not caused by an increase in LCB efflux or change in lipid composition. (**a**) BY4741 (wild-type; WT) and AOY13 (*faa1*Δ *faa4*Δ; ΔΔ) cells were labeled with [^3^H]DHS at 30 °C for 5 min. After being washed twice with medium containing BSA, cells with equal radioactivities were suspended in fresh medium containing BSA and incubated at 30 °C for 10 min. Radioactivities associated with cells and medium were counted by a liquid scintillation counter, and those associated with cells are expressed as a percentage of the total radioactivity. Values represent the means ± SDs of three independent experiments, and statistically significant differences are indicated (*t*-test; *p < 0.05). (**b**) BY4741, 1825 (*rsb1*Δ), AOY13, and TNY12 (*faa1*Δ *faa4*Δ *rsb1*Δ) were incubated with 20 μM [^3^H]DHS at 30 °C for 5 min, and transport activity was determined as in (**a**). Values represent the means ± SDs of three independent experiments, and statistically significant differences are indicated (*t*-test; **p < 0.01). (**c**) TNY34 (*faa4*Δ *FAA1-HA-AID*) cells were incubated with 500 μM IAA at 30 °C for 1 hr. Total cell lysates were prepared, separated by SDS-PAGE, and subjected to immunoblotting with anti-AID or, to demonstrate equal protein loading, anti-Pgk1 antibody. IB, immunoblotting. (**d**) CTY3 (wild-type), TNY32 (*faa4*Δ), and TNY34 cells were incubated with 500 μM IAA at 30 °C for 1 hr and then treated with 20 μM [^3^H]DHS for 5 min. Transport activity was determined as in (**a**). Values represent the means ± SDs of three independent experiments, and statistically significant differences are indicated (*t*-test; **p < 0.01).

In theory, it was possible that the apparent reduction in LCB uptake in *faa1*Δ *faa4*Δ cells was caused by increased efflux. Therefore, to exclude this possibility, we performed a LCB release assay using [^3^H]DHS. After cells were loaded with [^3^H]DHS for 1 hr, the amounts of [^3^H]DHS released for next 10 min were measured. Wild-type cells released 13% of the preloaded DHS (Fig. 2a). The amount of released DHS was slightly higher in *faa1*Δ *faa4*Δ cells (15% of preloaded [^3^H]DHS). However, this slight increase cannot explain the large reduction in the DHS uptake observed in *faa1*Δ *faa4*Δ cells (Fig. 1e). Rather, the difference in the released DHS amounts between wild-type and *faa1*Δ *faa4*Δ cells may have been caused by the differences in their import activities, since some fraction of the released DHS re-entered into cells during the incubation period.

We next examined the effect of *RSB1* deletion on LCB uptake. Rsb1 is a putative LCB efflux transporter, and LCB export is blocked by *RSB1* deletion^30^,^31^. The *rsb1*Δ mutation had no effect on DHS uptake when introduced into either wild-type or *faa1*Δ *faa4*Δ cells (Fig. 2b). This result again indicates that the observed decrease in LCB uptake in *faa1*Δ *faa4*Δ cells (Fig. 1e) is not caused by increased export but indeed by reduced import.

Type I FA synthase (FAS) is responsible for the majority of *de novo* FA synthesis. Mammalian FAS is a multifunctional enzyme and contains a thioesterase domain^32^. Therefore, the products of FAS are FAs (mainly palmitic acid), which must be activated by ACSs before utilization as the precursors for lipid synthesis. On the other hand, yeast FAS does not contain a thioesterase domain^33^. Accordingly, in yeast, acyl-CoA is released from FAS and utilized for subsequent metabolism without the aid of ACSs. Therefore, the contribution of lipids metabolized by yeast ACSs to cellular lipid composition is low. Indeed, a previous report demonstrated that FA profiles in *faa1*Δ *faa4*Δ cells are essentially unchanged compared with wild-type cells^6^. Furthermore, overall lipid compositions are almost indistinguishable between wild-type and *faa1*Δ *faa4*Δ cells (Supplementary Fig. 2). Thus, it is unlikely that the decreased LCB uptake in *faa1*Δ *faa4*Δ cells is caused by an indirect effect such as changes in lipid composition or membrane properties. Nonetheless, to further exclude this possibility, we utilized the transient protein degradation system called AID (auxin-inducible degron). In this system, treatment with the auxin 3-indoleacetic acid (IAA) causes rapid degradation of proteins fused with the AID tag^34^. We created *FAA1-AID faa4*Δ cells, in which the *FAA1* gene was chromosomally tagged with the *AID* sequence. We expected that the transient degradation of Faa1-AID achieved by this system would enable us to isolate any effects of Faa1 disappearance on LCB uptake while keeping changes in lipid composition to a minimum. Treatment of *faa4*Δ *FAA1-AID* cells with IAA indeed caused rapid degradation of Faa1-AID, although a small fraction of Faa1-AID still remained (Fig. 2c). IAA treatment did not affect DHS uptake in the control cells (Fig. 2d). On the other hand, it caused a significant decrease in DHS transport into *faa4*Δ *FAA1-AID* cells. The import defect was not as severe in *faa4*Δ *FAA1-AID* cells (Fig. 2d) compared with *faa1*Δ *faa4*Δ cells (Fig. 1e): this partial rescue of function may have been due to residual Faa1-AID protein in the *faa4*Δ *FAA1-AID* cells treated with IAA (Fig. 2c). These results exclude the possibility that changes in membrane composition indirectly affect LCB uptake in *faa1*Δ *faa4*Δ cells, and further support the notion that the ACSs themselves are directly involved in LCB import.

### Imports of LCFAs and LCBs are competitive

**Figure 3:**
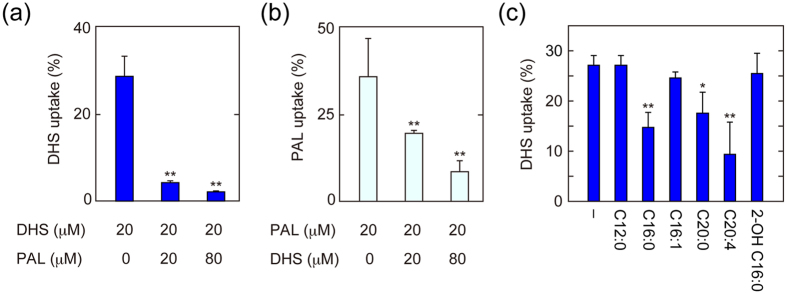
LCB and LCFA transports into cells are competitive with each other. (**a**–**c**) BY4741 (wild-type) cells were labeled with 20 μM [^3^H]DHS in the presence of cold palmitic acid (PAL) at the indicated concentration at 30 °C for 5 min (**a**). BY4741 cells were labeled with 20 μM [^3^H]palmitic acid in the presence of cold DHS at the indicated concentration at 30 °C for 30 min (**b**). BY4741 cells were labeled with 20 μM [^3^H]DHS in the presence of 20 μM cold FA as indicated (C12:0, lauric acid; C16:0, palmitic acid; C16:1, palmitoleic acid; C20:0, arachidic acid; C20:4, arachidonic acid; 2-OH C16:0, (+/−)-2-hydroxypalmitic acid) at 30 °C for 5 min (**c**). Radioactivities associated with cells, medium, and glass test tubes were counted by a liquid scintillation counter, and those associated with cells are expressed as a percentage of the total radioactivity. Values represent the means ± SDs of three independent experiments, and statistically significant differences are indicated (*t*-test; **p < 0.01).

Our findings indicate that Faa1 and Faa4 are involved in both LCFA and LCB uptakes. Under substrate competition, two (or more) substrates for the same enzyme compete with each other and are metabolized at difference rates depending on concentration and enzyme affinity. If Faa1 and Faa4 act as transporters both for LCFAs and LCBs, the transport of LCFAs should be competitively inhibited by LCBs, and *vice versa*. When a [^3^H]DHS uptake assay was conducted in the presence of palmitic acid, the imported DHS was reduced in a palmitic acid dose-dependent manner (Fig. 3a). Addition of palmitic acid at concentrations equal to and 4-fold that of DHS caused respective reductions in imported [^3^H]DHS by 85% and 92% (with respect to import levels in the absence of palmitic acid).

The uptake of [^3^H]palmitic acid was also inhibited by DHS addition (Fig. 3b). However, the competitive efficiency of DHS was lower than that of palmitic acid. Addition of DHS at concentrations equal to and 4-fold that of palmitic acid resulted in respective decreases in imported [^3^H]palmitic acid by 50% and 79% (with respect to import levels in the absence of DHS). These results indicate that Faa1 and Faa4 prefer palmitic acid as a transport substrate over DHS. The competition between palmitic acid and DHS in uptake suggests that they are imported into cells through the same transporters.

We next examined competitive inhibition of DHS uptake by other FAs. Palmitic acid and arachidonic acid (C20:4) inhibited DHS uptake (Fig. 3c). Arachidic acid (C20:0) also restricted uptake, albeit weakly. On the other hand, lauric acid (C12:0), palmitoleic acid (C16:1), and 2-hydroxypalmitic acid had no effect. Thus, the number of FA species that competitively interfered with DHS transport was limited.

### LCFA and LCB uptakes differ in their energy requirements

**Figure 4:**
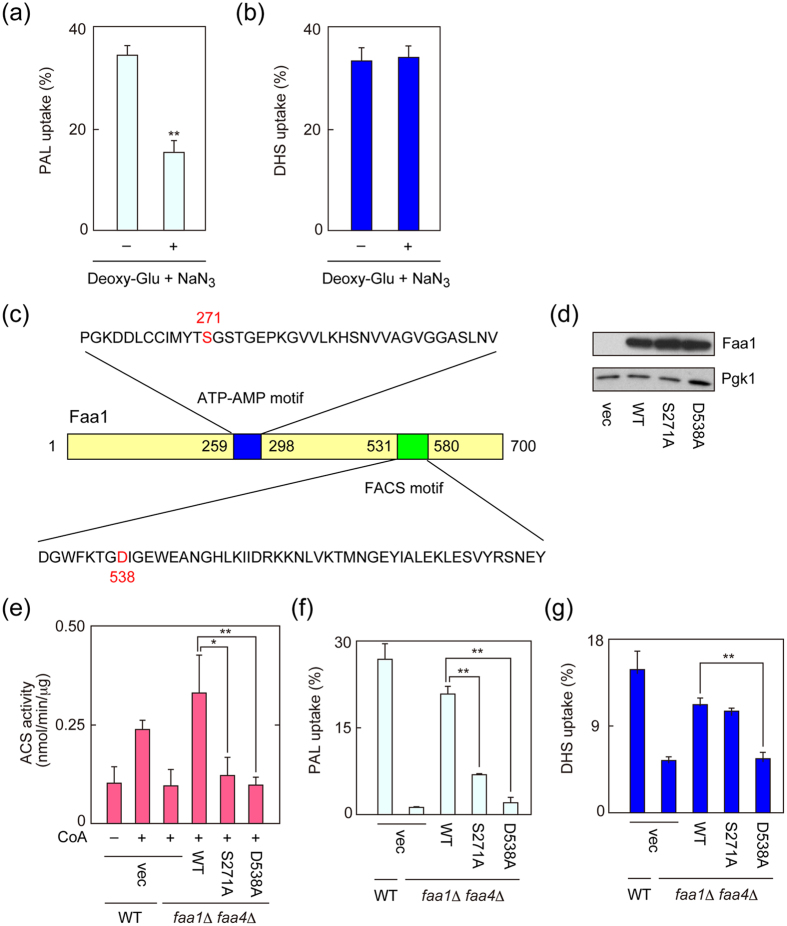
ATP-dependency is different between LCB and LCFA uptakes. (**a**,**b**) BY4741 (wild-type; WT) cells were incubated with 50 mM 2-deoxy-D-glucose (Deoxy-Glu) and 3 mM sodium azide (NaN_3_) at 30 °C for 10 min and then labeled with 20 μM [^3^H]palmitic acid (PAL) for 30 min (**a**) or 20 μM [^3^H]DHS for 5 min (**b**). Radioactivities associated with cells, medium, and glass test tubes were counted by a liquid scintillation counter, and those associated with cells are expressed as a percentage of the total radioactivity. Values represent the means ± SDs of three independent experiments, and statistically significant differences are indicated (*t*-test; **p < 0.01). (**c**) Structure of the Faa1 protein and the location and sequences of the ATP-AMP and FACS motifs are represented. The amino acid residues mutated in this study are shown in red. (**d**,**e**) AOY13 (*faa1*Δ *faa4*Δ) cells harboring the pAKNF316 (vector; vec), pNRT15 (*3xFLAG-FAA1*), pNRT39 (*3xFLAG-FAA1 S271A*), or pNRT40 (*3xFLAG-FAA1 D538A*) plasmid were grown in SC-URA medium at 30 °C. (**d**) Total cell lysates were prepared from them, separated by SDS-PAGE, and detected by immunoblotting with anti-DYKDDDDK or, to demonstrate equal protein loading, anti-Pgk1 antibody. (**e**) Total cell lysates prepared from them were subjected to an *in vitro* ACS assay using 10 μM [^3^H]palmitic acid in the presence or absence of CoA. The assay was performed at 37 °C for 2 min. The reaction product [^3^H]palmitoyl-CoA was separated from unreacted [^3^H]palmitic acid by phase-separation, and its radioactivity was measured using a liquid scintillation counter. Values represent the means ± SDs of three independent experiments, and statistically significant differences are indicated (*t*-test; *p < 0.05; **p < 0.01). (**f**,**g**) BY4741 cells bearing the pAKNF316 plasmid and AOY13 cells bearing the pAKNF316, pNRT15, pNRT39, or pNRT40 plasmid were labeled with 20 μM [^3^H]palmitic acid at 30 °C for 30 min (**f**) or with 20 μM [^3^H]DHS at 30 °C for 5 min (**g**). Transport activity was determined as in (**a**). Values represent the means ± SDs of three independent experiments, and statistically significant differences are indicated (*t*-test; **p < 0.01).

To gain insight into the energy requirements for LCB uptake, we performed a LCB uptake assay in the presence of sodium azide and 2-deoxy-D-glucose, which deplete cellular ATP. Treatment with sodium azide/2-deoxy-D-glucose inhibited palmitic acid uptake to 44% of levels observed in untreated cells (Fig. 4a). On the other hand, the same treatment had no effect on DHS transport into cells (Fig. 4b). These results indicate that ATP is needed for LCFA transport, but not for DHS import. Thus, although LCFA and DHS appear to be transported into cells via same transporters, their energy requirements are different.

Faa1 and Faa4 contain two highly conserved motifs, the ATP-AMP motif and FACS (fatty ACS) motif^5^,^35^ (Fig. 4c). The ATP-AMP motif exists in all adenylate families including the ACS family. The FACS motif has been proposed to be required for catalytic activity and substrate specificity in the ACS family based on mutational analyses using the *E. coli* ACS FadD^36^. We created Ala-substituted mutants (S271A and D538A) of the Faa1 protein. These sites, Ser271 and Asp538, are highly conserved residues among ACSs, and located in the ATP-AMP motif and FACS motif, respectively. In terms of gene expression, wild-type and mutant Faa1 proteins were expressed at similar levels (Fig. 4d). To investigate the effects of these mutations on ACS activity, we performed an *in vitro* ACS assay using [^3^H]palmitic acid as a substrate. The ACS activity of the Faa1 S271A protein was much lower than that of the wild-type protein (11% of wild-type activity, calculated after subtraction of the activity in vector-transfected cells here and hereafter; Fig. 4e). The ACS activity in *faa1*Δ *faa4*Δ cells expressing Faa1 D538A was nearly equal to that in cells harboring the vector, indicating that Faa1 D538A had almost no ACS activity. The palmitic acid transport activity of the Faa1 S271A protein was decreased to 29% that of the wild-type protein (Fig. 4f). In terms of transport activity as well, the Faa1 D538A protein again exhibited a severer defect (4% of the activity of the wild-type protein; Fig. 4f).

We next examined the effects of these mutations on DHS uptake. The amount of transported DHS in cells expressing Faa1 D538A was nearly identical to that in cells harboring the vector (Fig. 4g), indicating that Faa1 D538A had almost no DHS transport activity. Therefore, the ACS activity of Faa1 D538A was well correlated with both its palmitic acid and DHS transport activities. On the other hand, the DHS transport activity of Faa1 S271A was not correlated with its ACS activity. Although the Faa1 S271A had only residual ACS activity (Fig. 4e), its DHS transport activity was equivalent to that of the wild-type Faa1 protein (Fig. 4g). Since Ser271 is a well-conserved residue in the ATP-AMP motif, it is likely essential for ATP utilization. Together with the result obtained from sodium azide/2-deoxy-D-glucose treatment (Fig. 4a,b), these results indicate that DHS and palmitic acid transports are different in terms of their utilization of ATP (or lack thereof).

### Mammalian ACSLs are involved in LCB uptake

**Figure 5:**
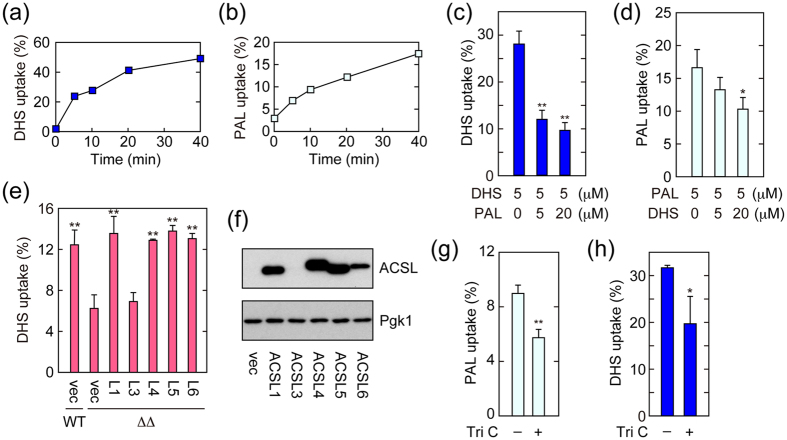
Mammalian ACSLs are involved in DHS uptake. (**a**,**b**) IEC-6 cells were incubated with 5 μM [^3^H]DHS (**a**) or [^3^H]palmitic acid (PAL) (**b**) for the indicated time period. Radioactivities associated with cells and medium were counted by a liquid scintillation counter, and those associated with cells are expressed as a percentage of the total radioactivity. (**c**,**d**) IEC-6 cells were labeled with 5 μM [^3^H]DHS (**c**) or 5 μM [^3^H] palmitic acid (PAL; **d**) in the presence of cold palmitic acid or DHS, respectively, at the indicated concentration at 37 °C for 15 min. Transport activity was determined as in (**a**). Values represent the means ± SDs of three independent experiments, and statistically significant differences are indicated (*t*-test; *p < 0.05; **p < 0.01). (**e**,**f**) BY4741 (wild-type; WT) or AOY13 (*faa1*Δ *faa4*Δ; ΔΔ) cells harboring the pAKNF316 (vector; vec), pAO15 (*3xFLAG-ACSL1*; L1), pAO16 (*3xFLAG-ACSL3*; L3), pAO17 (*3xFLAG-ACSL4*; L4), pAO19 (*3xFLAG-ACSL5*; L5), or pAO21 (*3xFLAG-ACSL6*; L6) plasmid were grown in SC-URA medium at 30 °C. (**e**) Cells were labeled with 20 μM [^3^H]DHS at 30 °C for 5 min. Radioactivities associated with cells, medium, and glass test tubes were counted by a liquid scintillation counter, and those associated with cells are expressed as a percentage of the total radioactivity. Values represent the means ± SDs of three independent experiments. Statistically significant differences with respect to the value of AOY13 (*faa1*Δ *faa4*Δ) cells harboring vector are indicated (*t*-test; **p < 0.01). (**f**) Total cell lysates were prepared, separated by SDS-PAGE, and subjected to immunoblotting with anti-FLAG or, to demonstrate equal protein loading, anti-Pgk1 antibody. (**g**,**h**) IEC-6 cells were treated with DMSO or 20 μM triacsin C (Tri C) at 37 °C for 1 hr. Cells were then labeled with 5 μM [^3^H] palmitic acid (**g**) or 5 μM [^3^H]DHS (**h**) at 37 °C for 15 min. Transport activity was determined as in (**a**). Values represent the means ± SDs of three independent experiments, and statistically significant differences are indicated (*t*-test; *p < 0.05; **p < 0.01).

We next expanded our analyses on LCB uptake from yeast to mammals. We first examined the time course of [^3^H]DHS and [^3^H]palmitic acid uptakes using the small intestine epithelial cells IEC-6. Both imports of [^3^H]DHS and [^3^H]palmitic acid gradually started to plateau around 10 min (Fig. 5a,b). We then investigated the competitive uptake of palmitic acid versus DHS. Import of [^3^H]palmitic acid was inhibited by DHS, and *vice versa* (Fig. 5c,d), suggesting existence of common transporter(s) for LCFAs and LCBs in mammals, as in yeast.

Yeast Faa1 and Faa4 share high sequence similarity with mammalian ACSL family members (ACSL1, 3, 4, 5, and 6). To examine the involvement of ACSLs in DHS uptake, each of the human ACSL family members was cloned into the yeast expression vector and expressed as 3xFLAG-tagged protein in *faa1*Δ *faa4*Δ cells. Separate introductions of *ACSL1*, *4*, *5*, and *6* recovered the deficient [^3^H]DHS uptake in *faa1*Δ *faa4*Δ cells (Fig. 5e). ACSL3 failed to be expressed in yeast (Fig. 5f): accordingly, the introduction of the *ACSL3* plasmid had no effect on [^3^H]DHS uptake (Fig. 5e). We also tested the DHS transport activities of other members of ACS subfamilies (ACSM and ACSVL). Among them, only ACSVL4 increased DHS uptake (Supplementary Fig. 3). It has been reported that ACSVL4 exhibits ACS activity not only toward VLCFAs but also toward LCFAs^37^,^38^.

We then examined the involvement of ACSLs in DHS uptake using IEC-6 cells and the ACSL inhibitor triacsin C. Triacsin C treatment caused reduced transports of both [^3^H]palmitic acid and [^3^H]DHS (Fig. 5g,h). These results indicate that the mammalian ACSs ACSLs and ACSVL4 are also involved in LCB uptake, in a similar way to their yeast counterparts.

## Discussion

Eukaryotic cells can import exogenous LCBs and utilize them as precursors for sphingolipids and glycerophospholipids. Since a LCB forms part of the hydrophobic backbone of sphingolipids, LCBs can be directly incorporated into sphingolipids during synthesis. Alternatively, LCBs can be metabolized to FAs via LCB 1-phosphates and fatty aldehydes then metabolized to glycerophospholipids^26^,^39^,^40^. Humans consume abundant dietary sphingolipids (300–400 mg per day)^17^. Some beneficial effects of dietary sphingolipids have been reported, such as reduced serum LDL cholesterol, prevention of colon carcinogenesis and inflammation, and improved skin barrier function^19^,^20^,^21^,^22^,^23^. Dietary sphingolipids enter into small-intestinal epithelial cells after degradation into FAs and LCBs^18^. Despite the nutritional importance of sphingolipids, the molecular mechanism by which LCBs are imported into cells has remained completely unknown. In the present study, we revealed that ACS family members (yeast Faa1 and Faa4 and mammalian ACSL1, ACSL4, ACSL5, ACSL6, and ACSVL4), which have been implicated in LCFA transport, are involved in LCB uptake as well (Figs 1 and 5; Supplementary Fig. 3). Furthermore, we also found that the transports of LCFAs and LCBs are mutually competitive (Figs 3 and 5), implying that dietary sphingolipids inhibit LCFA uptake. This inhibition is likely responsible for the tempering effect of dietary sphingolipid intake on serum LDL levels.

The most well-known role of ACS family proteins is to catalyze the conversion of a FA to its corresponding acyl-CoA, which step is necessary for the subsequent metabolism of the FA. On the other hand, some members of this family, such as ACSL1 and FATP1/ACSVL5, have been proposed to act as FA transporters^1^. Whether ACSs have transporter function or not is a hotly debated topic. Since both the metabolic function (addition of CoA) and transporter function of ACSs utilize LCFAs as the substrate, dissociating the two has been difficult to achieve experimentally. In one attempt to do so, Zou *et al.*^41^ performed mutational analyses on yeast Fat1^41^. Although most mutants exhibited similar reductions in both metabolic and transporter activities, some mutants showed partial dissociation of them. For example, while FA import activity was largely reduced in Fat1 F528A and Fat1 L669R mutants, their metabolic activity was only moderately decreased. On the other hand, Fat1 S258A and Fat1 D508A were essentially devoid of metabolic activity, but retained some LCFA import activity. Their results supported the idea that ACSs have both transporter and metabolic activities, but the separation of these two activities was partial, and the proposition of independent operation not convincing. In this respect, LCBs were ideal as target compounds: the LCBs used were not substrates of Faa1 and Faa4 for metabolic activity, but LCB uptake was still dependent on them. Thus, the use of LCBs allowed us to clearly dissociate the metabolic activities of Faa1 and Faa4 from their transporter activities. Our results strongly suggest that Faa1 and Faa4 have transporter functions, and hint at this possibility for other ACS family members as well.

Our findings revealed that the transports of LCFAs and LCBs differed in terms of their energy requirements. Although LCFA uptake was inhibited by sodium azide/2-deoxy-D-glucose, import of DHS was not (Fig. 4). Furthermore, the ATP-AMP motif mutant Faa1 S271A exhibited severe defects in LCFA transport as well as in ACS activity, whereas its LCB transport activity was nearly normal (Fig. 4). These results indicate that ATP is required for the transport of LCFAs but not for LCB influx. The exact reason why ATP dependency is different between LCFA and LCB uptakes is currently unclear: we speculate that the concentration gradient between the extracellular space and the intracellular fluid is the driving force behind both LCB and LCFA uptakes, and differences in these gradients are responsible for the distinct energy dependencies. Since the intracellular concentration of LCBs is quite low under physiological conditions, the difference between it and the extracellular concentrations used in this experiment may allow the import of LCBs without ATP. In contrast, the intracellular concentration (or more specifically, the local concentration at the inner leaflet of plasma membrane) of LCFAs seems to be much higher than that of LCB. Therefore, it is possible that conversion of LCFAs to their acyl-CoAs, which process requires ATP, is necessary to create a sufficient concentration gradient between the extracellular space and intracellular fluid.

In summary, here we revealed that ACSs (Faa1 and Faa4 in yeast and ACSL1, ACSL4, ACSL5, ACSL6, and ACSVL4 in mammals) are involved in LCB uptake. In addition, we provided important insight into the molecular mechanism of LCFA transport. Our findings strongly support the hypothesis that ACSs possess transporter activity, although this must be further verified with *in vitro* analyses using purified proteins. LCBs show great promise as useful tools for analyses into the transporter function(s) of ACSs.

## Methods

### Yeast strains and media

The *Saccharomyces cerevisiae* strain BY4741 (*MAT**a** his3*Δ*1 leu2*Δ*0 met15*Δ*0 ura3*Δ*0*)^42^ and its derivatives 6477 (*faa1*Δ*::KanMX4*), 833 (*faa4*Δ*::KanMX4*), 3178 (*fat1*Δ*::KanMX4*), and 1825 (*rsb1*Δ*::KanMX4*)^43^ were obtained from Open Biosystems (Huntsville, AL). AOY13 (BY4741, *faa1*Δ*::KanMX4 faa4*Δ*::NatNT2*) and CTY3 (BY4741, *ura3*Δ*0::pADH-OsTIR1-9xMyc-URA3*) have been described previously^26^,^44^. TNY12 (BY4741, *faa1*Δ*::KanMX4 faa4*Δ*::NatNT2 rsb1*Δ*::HIS3*) and TNY32 (BY4741, *ura3*Δ*0::pADH-OsTIR1-9xMyc-URA3 faa4*Δ*::NatNT2*) cells were constructed by introducing *rsb1*Δ*::HIS3* and *faa4*Δ*::NatNT2* mutations respectively into AOY13 and CTY3 cells by homologous recombination. TNY5 (BY4741, *fat1*Δ*::LEU2*) cells were constructed by replacing a *KanMX4* cassette in 3178 cells with the *LEU2* marker. TNY11 (BY4741, *faa1*Δ*::KanMX4 faa4*Δ*::NatNT2 fat1*Δ*::LEU2*) cells were constructed by introducing a *fat1*Δ*::LEU2* mutation into AOY13 cells by homologous recombination. TNY34 (TNY32, *FAA1-HA-AID::KanMX6*), TNY42 (BY4741, *FAA1-GFP::KanMX6*), TNY51 (BY4741, *FAA4-GFP::LEU2*) cells were constructed from TNY32 or BY4741 cells by chromosomal fusion of the *FAA1* or *FAA4* gene with *HA-AID* or with *GFP* as described previously^45^,^46^. Cells were grown in YPD medium (1% yeast extract, 2% peptone, and 2% D-glucose) or SC medium lacking uracil (SC-URA; 0.67% yeast nitrogen base, 2% D-glucose, 0.5% casamino acids, 20 mg/l adenine, and 20 mg/l tryptophan) at 30 °C.

### Cell culture

Rat small intestine epithelial cells (IEC-6 cell line^47^) were provided by the RIKEN BRC and grown in Dulbecco’s modified Eagle’s medium (DMEM; Sigma, St. Louis, USA) containing 10% FCS and supplemented with 100 U/ml penicillin, 100 μg/ml streptomycin (Sigma), and 4 μg/ml insulin (Wako Pure Chemical Industries, Osaka, Japan). Triacsin C was purchased from Alomone Labs (Jerusalem, Israel).

### Plasmids

The pAKNF316 plasmid (*URA3* marker; *CEN*) is a yeast expression vector and was designed to produce an N-terminal 3xFLAG-tagged protein from the promoter of the *TDH3* gene, which encodes glycerol-3-phosphate dehydrogenase (GAPDH). It was constructed by cloning the *TDH3* promoter^30^ and *3xFLAG* sequence into the pRS316 plasmid^48^. The *FAA1* gene was amplified by PCR from yeast genomic DNA using primers 5′-GCTAGC ATGGTTGCTCAATATACCGTTCCAG-3′ (*Nhe*I site underlined) and 5′-TTAAGACGAACTATAAACGGCGTCAAC-3′. The resulting DNA fragment was first cloned into the TA cloning vector pGEM-T Easy (Promega, Madison, WI), then the *Nhe*I-*Not*I fragment was transferred into the *Xba*I-*Not*I site of pAKNF316, generating the pNRT15 plasmid (*3xFLAG-FAA1*). The *FAA1* mutants were created using QuikChange site-directed mutagenesis kit (Agilent Technologies, Santa Clara, CA) with following primers: for *FAA1 S271A*, 5′-GTTGCATCATGTATACGGCTGGTTCTACAGGTGAG-3′ and 5′-CTCACCTGTAGAACCAGCCGTATACATGATGCAAC-3′; and for *FAA1 D538A*, 5′-GTTGGTTCAAGACCGGTGCCATCGGTGAATGGGAAG-3′ and 5′-CTTCCCATTCACCGATGGCACCGGTCTTGAACCAAC-3′. The pAO15 (*3xFLAG-ACSL1*), pAO16 (*3xFLAG-ACSL3*), pAO17 (*3xFLAG-ACSL4*), pAO19 (*3xFLAG-ACSL5*), pAO21 (*3xFLAG-ACSL6*), pAO26 (*3xFLAG-ACSM1*), pAO80 (*3xFLAG-ACSM2A*), pAO64 (*3xFLAG-ACSM2B*), pAO65 (*3xFLAG-ACSM3*), pAO66 (*3xFLAG-ACSM4*), pAO67 (*3xFLAG-ACSM5*), pAO27 (*3xFLAG-ACSVL1*), pAO68 (*3xFLAG-ACSVL2*), pAO69 (*3xFLAG-ACSVL3*), pAO70 (*3xFLAG-ACSVL4*), pAO71 (*3xFLAG-ACSVL5*), and pAO72 (*3xFLAG-ACSVL6*) plasmids have been described previously^27^.

### Lipid uptake assay

Radiolabeled lipids used were [4,5-^3^H]DHS (60 Ci/mmol; American Radiolabeled Chemical, St. Louis, USA), [3-^3^H]SPH (20 Ci/mmol; American Radiolabeled Chemical), [11,12-^3^H]PHS (40 Ci/mmol)^40^, and [9,10-^3^H]palmitic acid (60 Ci/mmol; American Radiolabeled Chemical). Stock solutions of each lipid having equal concentration and radioactivity (1.5 mM and 0.014 μCi/μl for labeling of yeast cells; 0.4 mM and 0.017 μCi/μl for labeling of mammalian cells) were prepared by mixing each radioactive lipid and its corresponding cold lipid (DHS, Biomol, Plymouth Meeting, USA; PHS, Enzo Life Sciences, Farmingdale, USA; SPH, Biomol; palmitic acid, Sigma) in ethanol. Yeast cells (1 × 10^7^ cells/ml) in 0.5 ml of YPD or SC-URA medium were incubated with 7 μl of lipid solution (0.1 μCi and final concentration of 20 μM) at 30 °C. Cells were chilled on ice, and medium (fraction I) was separated from cells by centrifugation (2,000 g, 4 °C, 3 min). Cells were washed with 0.5 ml of cold medium containing 1 mg/ml of FA-free BSA (A6003; Sigma). After removal of medium (fraction II) by centrifugation, cells were suspended in 0.5 ml of cold medium and transferred from glass test tubes to microcentrifuge tubes. Cells were recovered by centrifugation and suspended in 100 μl of lipid extraction solution (ethanol/water/diethyl ether/pyridine/15 N ammonia (15:15:5:1:0.018, v/v). Lipids were extracted by incubating at 60 °C for 15 min with mixing at 5-min intervals. The extraction step was repeated again, and the extracted lipids were pooled (fraction III). Lipids absorbed to glass tubes used for the labeling experiments were extracted with 0.2 ml of ethanol (fraction IV). Radioactivity associated with each fraction was measured using a liquid scintillation counter (LSC-3600; Aloka, Tokyo, Japan). Transport activity was calculated as radioactivity in fraction III per total radioactivities (radioactivities in fractions I+II+III+IV) and expressed as a percentage. Lipid competition experiments were performed by labeling cells with 20 μM [^3^H]DHS or [^3^H]palmitic acid in the presence of 20 μM or 80 μM cold DHS (Biomol), palmitic acid (Sigma), lauric acid (Sigma), palmitoleic acid (Sigma), arachidic acid (Sigma), arachidonic acid (Sigma), or (+/−)-2-hydroxypalmitic acid (Wako Pure Chemical Industries, Osaka, Japan).

Lipid uptake assay using IEC-6 cells grown on 12-well dishes was performed as described below. Culture medium was changed to 0.5 ml of DMEM without FCS but containing 4 μg/ml insulin. After one hour of incubation at 37 °C, cells were treated with 6 μl of lipid solution (0.1 μCi and final concentration of 5 μM) at 37 °C for appropriate time periods. Cells were chilled on ice, and medium was removed. Cells were washed with 0.5 ml of cold PBS containing 1 mg/ml of FA-free BSA, suspended in 0.5 ml of the same solution, removed from dishes using scrapers, transferred to plastic tubes, and precipitated by centrifugation (750 g, 4 °C, 3 min). Lipids were extracted by suspending cells in 120 μl of chloroform/methanol (2:1, v/v). Radioactivities associated with medium and cell fractions were measured using the liquid scintillation counter LSC-3600. Transport activity was calculated as radioactivity in cell fraction per total radioactivities and expressed as a percentage.

### DHS release assay

Yeast cells were labeled with [^3^H]DHS (0.1 μCi, 3.3 nM) at 30 °C for 5 min. Cells were then chilled on ice, washed with cold YPD medium containing 1 mg/ml of BSA twice, and suspended in cold YPD medium containing 1 mg/ml of BSA. Cells with equal amount of radioactivity were incubated at 30 °C for 10 min. Cells (fraction A) were then chilled on ice and separated from medium (fraction B) by centrifugation. Radioactivities in fractions A and B were measured using the liquid scintillation counter LSC-3600. The percentage of released DHS for each sample was calculated as (radioactivity in fraction B)/[total radioactivities (radioactivities in fractions A+B)] × 100.

### [^3^H]DHS labeling assay

[^3^H]DHS labeling assay was performed as described previously^26^,^49^.

### Immunoblotting

Immunoblotting was performed as described previously^50^ using anti-DYKDDDDK (FLAG) (0.5 μg/ml; Wako Pure Chemical Industries), anti-FLAG M2 (1 μg/ml; Agilent Technologies), anti-AID (1:1000 dilution; BioROIS, Tokyo, Japan), or anti-Pgk1 antibody (1 μg/ml; Thermo Fisher Scientific, Waltham, USA) as the primary antibodies, and HRP-conjugated anti-mouse or anti-rabbit IgG F(ab’)_2_ fragment (each 1:7500 dilution; GE Healthcare Life Sciences, Buckinghamshire, UK) as the secondary antibodies. Detection was performed using Pierce ECL Western Blotting Substrate (Thermo Fisher Scientific) or Western Lightning Plus-ECL (PerkinElmer Life Sciences, Waltham, USA).

### *In vitro* ACS assay

Total membrane fractions were prepared as described previously^40^. Total membrane fractions (0.2 μg) were incubated with 10 μM [^3^H]palmitic acid at 37 °C for 2 min in 100 μl of ACS buffer [200 mM Tris-HCl (pH 7.5), 2.5 mM ATP, 8 mM MgCl_2_, 2 mM EDTA, 20 mM NaF, 0.1% Triton X-100, and 0.5 mM CoA]. The reaction was terminated by adding 500 μl of isopropanol/n-heptane/1 M H_2_SO_4_ (40:10:1, v/v). To remove unreacted [^3^H]palmitic acid, extraction by n-heptane was performed three times. In each step, 500 μl of n-heptane was added to the samples, then the organic phase (containing [^3^H]palmitic acid) was separated from the aqueous phase (containing the reaction product [^3^H]palmitoyl-CoA) by centrifugation (17,000 g, 4 °C, 5 min). Finally, radioactivity associated with the resulting aqueous phase was measured using the liquid scintillation counter LSC-3600.

## Additional Information

**How to cite this article**: Narita, T. *et al.* Long-chain bases of sphingolipids are transported into cells via the acyl-CoA synthetases. *Sci. Rep.* **6**, 25469; doi: 10.1038/srep25469 (2016).

## Supplementary Material

Supplementary Information

## References

[b1] Schaffer J. E. & Lodish H. F. Expression cloning and characterization of a novel adipocyte long-chain fatty-acid transport protein. Cell. 79, 427–436 (1994). 7954810 10.1016/0092-8674(94)90252-6

[b2] Watkins P. A., Maiguel D., Jia Z. & Pevsner J. Evidence for 26 distinct acyl-coenzyme A synthetase genes in the human genome. J Lipid Res. 48, 2736–2750 (2007). 17762044 10.1194/jlr.M700378-JLR200

[b3] Watkins P. A. Very-long-chain acyl-CoA synthetases. J Biol Chem. 283, 1773–1777 (2008). 18024425 10.1074/jbc.R700037200

[b4] Coe N. R., Smith A. J., Frohnert B. I., Watkins P. A. & Bernlohr D. A. The fatty acid transport protein (FATP1) is a very long chain acyl-CoA synthetase. J Biol Chem. 274, 36300–36304 (1999). 10593920 10.1074/jbc.274.51.36300

[b5] Black P. N. & DiRusso C. C. Yeast acyl-CoA synthetases at the crossroads of fatty acid metabolism and regulation. Biochim Biophys Acta. 1771, 286–298 (2007). 16798075 10.1016/j.bbalip.2006.05.003

[b6] Færgeman N. J., Black P. N., Zhao X. D., Knudsen J. & DiRusso C. C. The Acyl-CoA synthetases encoded within *FAA1* and *FAA4* in *Saccharomyces cerevisiae* function as components of the fatty acid transport system linking import, activation, and intracellular utilization. J Biol Chem. 276, 37051–37059 (2001). 11477098 10.1074/jbc.M100884200

[b7] Watkins P. A. *et al.* Disruption of the *Saccharomyces cerevisiae FAT1* gene decreases very long-chain fatty acyl-CoA synthetase activity and elevates intracellular very long-chain fatty acid concentrations. J Biol Chem. 273, 18210–18219 (1998). 9660783 10.1074/jbc.273.29.18210

[b8] Abumrad N., Coburn C. & Ibrahimi A. Membrane proteins implicated in long-chain fatty acid uptake by mammalian cells: CD36, FATP and FABPm. Biochim Biophys Acta. 1441, 4–13 (1999). 10526223 10.1016/s1388-1981(99)00137-7

[b9] Kihara A., Mitsutake S., Mizutani Y. & Igarashi Y. Metabolism and biological functions of two phosphorylated sphingolipids, sphingosine 1-phosphate and ceramide 1-phosphate. Prog Lipid Res. 46, 126–144 (2007). 17449104 10.1016/j.plipres.2007.03.001

[b10] Kihara A. & Igarashi Y. Production and release of sphingosine 1-phosphate and the phosphorylated form of the immunomodulator FTY720. Biochim Biophys Acta. 1781, 496–502 (2008). 18555808 10.1016/j.bbalip.2008.05.003

[b11] Mizutani Y., Mitsutake S., Tsuji K., Kihara A. & Igarashi Y. Ceramide biosynthesis in keratinocyte and its role in skin function. Biochimie. 91, 784–790 (2009). 19364519 10.1016/j.biochi.2009.04.001

[b12] Pontier S. M. & Schweisguth F. Glycosphingolipids in signaling and development: from liposomes to model organisms. Dev Dyn. 241, 92–106 (2012). 22038940 10.1002/dvdy.22766

[b13] Mitsutake S. & Igarashi Y. Sphingolipids in lipid microdomains and obesity. Vitam Horm. 91, 271–284 (2013). 23374721 10.1016/B978-0-12-407766-9.00012-2

[b14] Sassa T. *et al.* Impaired epidermal permeability barrier in mice lacking *Elovl1*, the gene responsible for very-long-chain fatty acid production. Mol Cell Biol. 33, 2787–2796 (2013). 23689133 10.1128/MCB.00192-13 PMC3700134

[b15] Ohno Y. *et al.* Essential role of the cytochrome P450 CYP4F22 in the production of acylceramide, the key lipid for skin permeability barrier formation. Proc Nat Acad Sci USA 112, 7707–7712 (2015). 26056268 10.1073/pnas.1503491112 PMC4485105

[b16] Dickson R. C., Sumanasekera C. & Lester R. L. Functions and metabolism of sphingolipids in *Saccharomyces cerevisiae*. Prog Lipid Res. 45, 447–465 (2006). 16730802 10.1016/j.plipres.2006.03.004

[b17] Vesper H. *et al.* Sphingolipids in food and the emerging importance of sphingolipids to nutrition. J Nutr. 129, 1239–1250 (1999). 10395583 10.1093/jn/129.7.1239

[b18] Kono M. *et al.* Neutral ceramidase encoded by the *Asah2* gene is essential for the intestinal degradation of sphingolipids. J Biol Chem. 281, 7324–7331 (2006). 16380386 10.1074/jbc.M508382200

[b19] Imaizumi K., Tominaga A., Sato M. & Sugano M. Effects of dietary sphingolipids on levels of serum and liver lipids in rats. Nutr Res. 12, 543–548 (1992).

[b20] Dillehay D. L., Webb S. K., Schmelz E. M. & Merrill A. H. Jr. Dietary sphingomyelin inhibits 1,2-dimethylhydrazine-induced colon cancer in CF1 mice. J Nutr. 124, 615–620 (1994). 8169652 10.1093/jn/124.5.615

[b21] Schmelz E. M. *et al.* Sphingomyelin consumption suppresses aberrant colonic crypt foci and increases the proportion of adenomas versus adenocarcinomas in CF1 mice treated with 1,2-dimethylhydrazine: implications for dietary sphingolipids and colon carcinogenesis. Cancer Res. 56, 4936–4941 (1996). 8895747

[b22] Kobayashi T., Shimizugawa T., Osakabe T., Watanabe S. & Okuyama H. A long-term feeding of sphingolipids affected the levels of plasma cholesterol and hepatic triacylglycerol but tissue phopholipids and sphingolipids. Nutr Res. 17, 111–114 (1997).

[b23] Duan J., Sugawara T., Sakai S., Aida K. & Hirata T. Oral glucosylceramide reduces 2,4-dinitrofluorobenzene induced inflammatory response in mice by reducing TNF-α levels and leukocyte infiltration. Lipids 46, 505–512 (2011). 21222241 10.1007/s11745-010-3518-9

[b24] Zhao Y. *et al.* Intracellular generation of sphingosine 1-phosphate in human lung endothelial cells: role of lipid phosphate phosphatase-1 and sphingosine kinase 1. J Biol Chem. 282, 14165–14177 (2007). 17379599 10.1074/jbc.M701279200 PMC2659598

[b25] Sassa T., Wakashima T., Ohno Y. & Kihara A. Lorenzo’s oil inhibits ELOVL1 and lowers the level of sphingomyelin with a saturated very long-chain fatty acid. J Lipid Res. 55, 524–530 (2014). 24489110 10.1194/jlr.M044586 PMC3934736

[b26] Nakahara K. *et al.* The Sjögren-Larsson syndrome gene encodes a hexadecenal dehydrogenase of the sphingosine 1-phosphate degradation pathway. Mol Cell. 46, 461–471 (2012). 22633490 10.1016/j.molcel.2012.04.033

[b27] Ohkuni A., Ohno Y. & Kihara A. Identification of acyl-CoA synthetases involved in the mammalian sphingosine 1-phosphate metabolic pathway. Biochem Biophys Res Commun. 442, 195–201 (2013). 24269233 10.1016/j.bbrc.2013.11.036

[b28] Tani M., Kihara A. & Igarashi Y. Rescue of cell growth by sphingosine with disruption of lipid microdomain formation in *Saccharomyces cerevisiae* deficient in sphingolipid biosynthesis. Biochem J. 394, 237–242 (2006). 16225461 10.1042/BJ20051354 PMC1386021

[b29] Wakashima T., Abe K. & Kihara A. Dual functions of the *trans*-2-enoyl-CoA reductase TER in the sphingosine 1-phosphate metabolic pathway and in fatty acid elongation. J Biol Chem. 289, 24736–24748 (2014). 25049234 10.1074/jbc.M114.571869 PMC4155643

[b30] Kihara A. & Igarashi Y. Identification and characterization of a *Saccharomyces cerevisiae gene*, *RSB1*, involved in sphingoid long-chain base release. J Biol Chem. 277, 30048–30054 (2002). 12034738 10.1074/jbc.M203385200

[b31] Kihara A. & Igarashi Y. Cross talk between sphingolipids and glycerophospholipids in the establishment of plasma membrane asymmetry. Mol Biol Cell. 15, 4949–4959 (2004). 15342785 10.1091/mbc.E04-06-0458 PMC524749

[b32] Smith S., Witkowski A. & Joshi A. K. Structural and functional organization of the animal fatty acid synthase. Prog Lipid Res. 42, 289–317 (2003). 12689621 10.1016/s0163-7827(02)00067-x

[b33] Lomakin I. B., Xiong Y. & Steitz T. A. The crystal structure of yeast fatty acid synthase, a cellular machine with eight active sites working together. Cell. 129, 319–332 (2007). 17448991 10.1016/j.cell.2007.03.013

[b34] Nishimura K., Fukagawa T., Takisawa H., Kakimoto T. & Kanemaki M. An auxin-based degron system for the rapid depletion of proteins in nonplant cells. Nat Methods 6, 917–922 (2009). 19915560 10.1038/nmeth.1401

[b35] Black P. N. & DiRusso C. C. Transmembrane movement of exogenous long-chain fatty acids: proteins, enzymes, and vectorial esterification. Microbiol Mol Biol Rev. 67, 454–472 (2003). 12966144 10.1128/MMBR.67.3.454-472.2003 PMC193871

[b36] Black P. N., Zhang Q., Weimar J. D. & DiRusso C. C. Mutational analysis of a fatty acyl-coenzyme A synthetase signature motif identifies seven amino acid residues that modulate fatty acid substrate specificity. J Biol Chem. 272, 4896–4903 (1997). 9030548 10.1074/jbc.272.8.4896

[b37] Herrmann T. *et al.* Mouse fatty acid transport protein 4 (FATP4): characterization of the gene and functional assessment as a very long chain acyl-CoA synthetase. Gene. 270, 31–40 (2001). 11404000 10.1016/s0378-1119(01)00489-9

[b38] Hall A. M., Wiczer B. M., Herrmann T., Stremmel W. & Bernlohr D. A. Enzymatic properties of purified murine fatty acid transport protein 4 and analysis of acyl-CoA synthetase activities in tissues from FATP4 null mice. J Biol Chem. 280, 11948–11954 (2005). 15653672 10.1074/jbc.M412629200

[b39] Kihara A. Sphingosine 1-phosphate is a key metabolite linking sphingolipids to glycerophospholipids. Biochim Biophys Acta. 1841, 766–772 (2014). 23994042 10.1016/j.bbalip.2013.08.014

[b40] Kondo N. *et al.* Identification of the phytosphingosine metabolic pathway leading to odd-numbered fatty acids. Nat Commun. 5, 5338 (2014). 25345524 10.1038/ncomms6338

[b41] Zou Z., DiRusso C. C., Ctrnacta V. & Black P. N. Fatty acid transport in *Saccharomyces cerevisiae*. Directed mutagenesis of *FAT1* distinguishes the biochemical activities associated with Fat1p. J Biol Chem. 277, 31062–31071 (2002). 12052836 10.1074/jbc.M205034200

[b42] Brachmann C. B. *et al.* Designer deletion strains derived from *Saccharomyces cerevisiae* S288C: a useful set of strains and plasmids for PCR-mediated gene disruption and other applications. Yeast. 14, 115–132 (1998). 9483801 10.1002/(SICI)1097-0061(19980130)14:2<115::AID-YEA204>3.0.CO;2-2

[b43] Winzeler E. A. *et al.* Functional characterization of the *S. cerevisiae* genome by gene deletion and parallel analysis. Science. 285, 901–906 (1999). 10436161 10.1126/science.285.5429.901

[b44] Tanaka C. *et al.* Hrr25 triggers selective autophagy-related pathways by phosphorylating receptor proteins. J Cell Biol. 207, 91–105 (2014). 25287303 10.1083/jcb.201402128 PMC4195827

[b45] Obara K., Yamamoto H. & Kihara A. Membrane protein Rim21 plays a central role in sensing ambient pH in *Saccharomyces cerevisiae*. J Biol Chem. 287, 38473–38481 (2012). 23019326 10.1074/jbc.M112.394205 PMC3493892

[b46] Obara K. & Kihara A. Signaling events of the Rim101 pathway occur at the plasma membrane in a ubiquitination-dependent manner. Mol Cell Biol. 34, 3525–3534 (2014). 25002535 10.1128/MCB.00408-14 PMC4135627

[b47] Quaroni A., Wands J., Trelstad R. L. & Isselbacher K. J. Epithelioid cell cultures from rat small intestine. Characterization by morphologic and immunologic criteria. J Cell Biol. 80, 248–265 (1979). 88453 10.1083/jcb.80.2.248 PMC2110349

[b48] Sikorski R. S. & Hieter P. A system of shuttle vectors and yeast host strains designed for efficient manipulation of DNA in *Saccharomyces cerevisiae*. Genetics. 122, 19–27 (1989). 2659436 10.1093/genetics/122.1.19 PMC1203683

[b49] Uemura S., Kihara A., Inokuchi J. & Igarashi Y. Csg1p and newly identified Csh1p function in mannosylinositol phosphorylceramide synthesis by interacting with Csg2p. J Biol Chem. 278, 45049–45055 (2003). 12954640 10.1074/jbc.M305498200

[b50] Kitamura T., Takagi S., Naganuma T. & Kihara A. Mouse aldehyde dehydrogenase ALDH3B2 is localized to lipid droplets via two C-terminal tryptophan residues and lipid modification. Biochem J. 465, 79–87 (2015). 25286108 10.1042/BJ20140624

