# Supplementary material for: Long-chain bases of sphingolipids are transported into cells via the acyl-CoA synthetases
Source: Sci Rep. 2016 May 3;6:25469. doi: 10.1038/srep25469 (PMC4853782; doi:10.1038/srep25469)

Long-chain bases of sphingolipids are transported into cells via the acyl-CoA synthetases

Tomomi Narita, Tatsuro Naganuma, Yurie Sase & Akio Kihara

Supplementary Figure 1 | Fat1 is not involved in DHS uptake. BY4741, TNY5 (*fat1*Δ), AOY13 (*faa1*Δ *faa4*Δ), and TNY11 (*faa1*Δ *faa4*Δ *fat1*Δ) cells were labeled with 20 μM [^{3}H]palmitic acid for 30 min (**a**) or 20 μM [^{3}H]DHS for 5 min (**b**). Radioactivities associated with cells, medium, and glass test tubes were counted by a liquid scintillation counter, and those associated with cells are expressed as a percent of the total radioactivity. Values represent the means ± SDs of three independent experiments, and statistically significant differences are indicated (*t*-test; **, $p < 0.01$).

Supplementary Figure 2 | Lipid profile of *faa1*Δ *faa4*Δ cells is indistinguishable from that of wild-type cells. Lipids were extracted from BY4741 (wild-type) and AOY13 (*faa1*Δ *faa4*Δ) cells, and those prepared from 1.4 OD_{600} cells were separated by normal-phase TLC and stained with cupric acetate/phosphoric acid solution. TG, triglyceride; Cer, ceramide; PE, phosphatidylethanolamine; PC, phosphatidylcholine; PS, phosphatidylserine; PI, phosphatidylinositol; IPC, inositol phosphorylceramide; MIPC, mannosylinositol phosphorylceramide; $M(IP)_2C$, mannosyldiinositol phosphorylceramide.

Supplementary Figure 3 | ACSVL4 is active in DHS uptake. (**a**, **b**) AOY13 (*faa1*Δ *faa4*Δ) cells harboring the pAKNF316 (vector; vec), pAO26 (*3xFLAG-ACSM1*), pAO80 (*3xFLAG-ACSM2A*), pAO64 (*3xFLAG-ACSM2B*), pAO65 (*3xFLAG-ACSM3*), pAO66 (*3xFLAG-ACSM4*), pAO67 (*3xFLAG-ACSM5*), pAO27 (*3xFLAG-ACSVL1*), pAO68 (*3xFLAG-ACSVL2*), pAO69 (*3xFLAG-ACSVL3*), pAO70 (*3xFLAG-ACSVL4*), pAO71 (*3xFLAG-ACSVL5*), or pAO72 (*3xFLAG-ACSVL6*) plasmid (**a**, **b**) and BY4741 (wild-type) cells harboring the pAKNF316 plasmid (**b**) were grown in SC-URA medium at 30 °C. (**a**) Total cell lysates were prepared, separated by SDS-PAGE, and detected by immunoblotting with anti-FLAG or anti-Pgk1 (loading control) antibody. (**b**) Cells were labeled with 20 μM

[^{3}H]DHS at 30 °C for 5 min. Radioactivities associated with cells, medium, and glass test tubes were measured by liquid scintillation counter and totaled, and those associated with cells are expressed as a percentage of total radioactivity.

Supplementary Figure 1

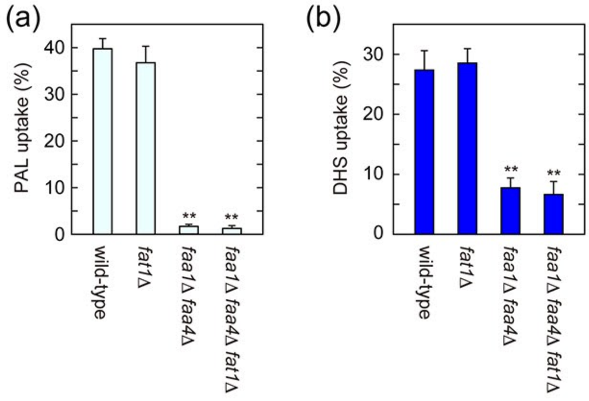

Supplementary Figure 2

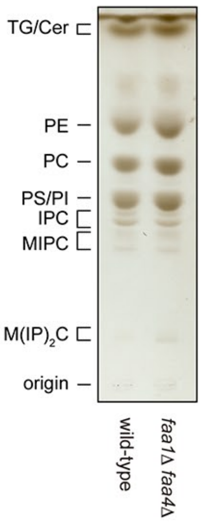

Supplementary Figure 3

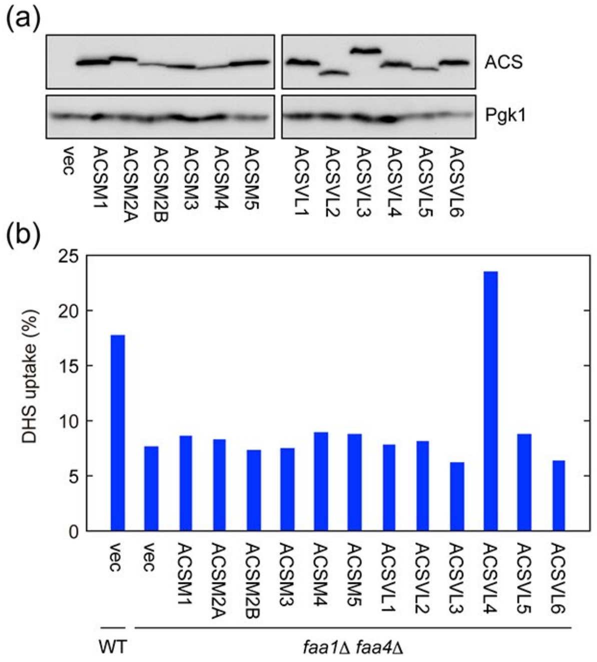

Supplement: Supplementary Information [file srep25469-s1.pdf]
